# Editorial: Strategies for functional polymeric nanocomposites and its multifunctional applications

**DOI:** 10.3389/fchem.2023.1274460

**Published:** 2023-08-17

**Authors:** Qinqi Wang, Adhimoorthy Prasannan, Nimita Jebaranjitham J., Leijiao Li, Wenliang Li

**Affiliations:** ^1^ School of Chemistry and Environmental Engineering, Changchun University of Science and Technology, Changchun, China; ^2^ Department of Materials Science and Engineering, National Taiwan University of Science and Technology, Taipei, Taiwan; ^3^ Department of Chemistry, Women’s Christian College (An Autonomous Institution Affiliated to University of Madras), Chennai, Tamil Nadu, India

**Keywords:** polymeric nanocomposite, wound repair, dendrimers drug-delivery systems, optical polymers, multifunctional applications

## 1 Introduction

The development of nanomaterial’s has greatly contributed to the development of modern medicine and optics, and polymers are an important support and branch of nanomaterial’s. They are non-toxic and harmless, can be degraded into small fragments in the body, and are finally excreted without causing inflammation. Due to their many advantages, biodegradable polymers have attracted more and more attention. In this Research Topic, we focus on reviewing and introducing some polymers for wound repair, drug loading or with special optical properties. These articles are categorized into three themes: 1) polydopamine for wound repair; 2) dendrimers as drug carriers; 3) optical polymers.

## 2 Polydopamine for wound repair

Polydopamine (PDA) is an important class of biomimetic polymers, which has good application prospects in the field of biomedicine. This subtopic covers a review of advances in the application of polydopamine (PDA) in the field of wound repair. PDA is a competitive candidate for wound dressings due to its excellent biocompatibility and biomimetic three-dimensional porous structure.

Herein, Cui et al. reviewed the application and development of PDA for wound hemostasis, healing and infection treatment in recent years. PDA has strong absorption in the near-infrared light region, which can convert light energy into heat energy and realize photothermal therapy for sterilization. The catechol group in PDA has good coordination and chelation ability and can coordinate with many metal ions, such as Fe^3+^, Ag^+^ and so on. NIR-induced metal-coordinated PDA composites produce peroxidase-like (POD)-like activity, which generates hydroxyl radicals (•OH) and releases metal ions, exhibiting excellent antibacterial properties. The catechol group in PDA interacts with the reactive residues of proteins or polysaccharides in blood, which can accelerate blood coagulation and achieve hemostasis. In addition, the combination of catechol group proteins can form a protective physical barrier to prevent the invasion of foreign microorganisms, provide protection for wounds, and at the same time inhibit the growth of bacteria and play an antibacterial effect. It can promote the transformation of macrophages from M1 type to M2 type, and has excellent anti-inflammatory, antibacterial, antioxidant, hemostatic properties and biocompatibility. Studies have shown that PDA coating can induce synergistic interactions of interface components, modify the interface properties of inorganic nanoparticles, and improve the adhesion of the material to platelets and red blood cells.

## 3 Dendrimers as drug carriers

Polymers are the cornerstone of various sustained-release and controlled-release drugs. Furosemide (FRSD) is a loop diuretic (4-chloro- n -furyl-5-sulfamoylaminobenzoic acid) used orally for the treatment of edema associated with cardiac, renal, and hepatic failure, and hypertension. According to the BCS classification system, FRSD has low oral bioavailability and solubility. It is rapidly absorbed from the gastrointestinal tract with a half-life of 30–120 min. Its bioavailability is about 60%–70%, but its absorption is variable and unstable. Murugan et al. synthesized two quaternary ammonium-based polyamidoamine dendrimers and used them as drug carriers to load FRSD. The surface amine modification and internal quaternization of PAMAM dendrimers played an important role in improving the dendrimer solubility and drug release. Moreover, the cytotoxicity is significantly reduced and the biocompatibility is improved. Therefore, the development of quaternary ammonium polyamidoamine dendrimers can provide new ideas for the development of new dendrimer drug delivery platforms in the future.

## 4 Optical polymers

Porphyrin-based polymers are excellent non-linear optical (NLO) materials due to their unique conjugated structure, good thermal stability, and narrow band gap. Liang et al. constructed a flurry of original porphyrin-based polymers covalently functionalized g-C_3_N_4_ nanohybrids through click chemistry between porphyrin-based polymers with alkyne end-groups [(PPorx-C≡CH (x = 1, 2 and 3)] and azide-functionalized graphitic carbon nitride (g-C_3_N_4_-N3), which was nominated as PPorx-g-C_3_N_4_ (x = 1, 2 and 3). Due to the photoinduced electron transfer (PET) between porphyrin-based polymers [PPorx (x = 1, 2 and 3)] group and graphite phase carbon nitride (g-C_3_N_4_) group in PPorx-g-C_3_N_4_ nanohybrids, the PPorx-g-C_3_N_4_ nanohybrids exhibited better non-linear optical (NLO) performance than the corresponding PPorx-C≡CH and g-C_3_N_4_-N3. It found that the imaginary third-order susceptibility (Im [*χ* ^(3)^]) value of the nanohybrids with different molecular weight (MW) of the PPorx group in the nanohybrids ranged from 2.5 × 10^3^ to 7.0 × 103 g mol^−1^ was disparate. Quite interestingly, the Im [*χ* ^(3)^] value of the nanohybrid with a PPorx group’s MW of 4.2 × 10^3^ g mol^−1^ (PPor2-g-C_3_N_4_) was 1.47 × 10^−10^ esu, which exhibited the best NLO performance in methyl methacrylate (MMA) of all nanohybrids. The PPorx-g-C_3_N_4_ was dispersed in polymethyl methacrylate (PMMA) to prepare the composites PPorx-g-C_3_N_4_/PMMA since PMMA was widely used as an alternative to glass. It suggested that PPorx-g-C_3_N_4_ nanohybrids were potential outstanding NLO materials. This study provided a new strategy for the design of porphyrin-based nanohybrids, which could find wider applications in the field of NLO materials.

## 5 Conclusion

In this Research Topic, a Research Topic of articles on different kinds of polymers for skin wound repair, drug delivery and optical materials. These articles describe recent advances in polymer-based materials for therapeutics and synthesis of optical materials.

